# Association Between Preoperative Diabetes Control and Postoperative Adverse Events Among Veterans Health Administration Patients With Diabetes Who Underwent Elective Ambulatory Hernia Surgery

**DOI:** 10.1001/jamanetworkopen.2023.6318

**Published:** 2023-03-31

**Authors:** Jessica Shanahan, Varsha G. Vimalananda, Laura Graham, Roman Schumann, Hillary J. Mull

**Affiliations:** 1 VA Boston Healthcare System, Boston, Massachusetts; 2 Department of Anesthesiology, Tufts University School of Medicine, Boston, Massachusetts; 3 Center for Healthcare Organization and Implementation Research, Bedford VA Medical Center, Bedford, Massachusetts; 4 Department of Medicine, Boston University Chobanian & Avedisian School of Medicine, Boston, Massachusetts; 5 Health Economics Resource Center, Palo Alto, Veterans Affairs Medical Center, Palo Alto, California; 6 Department of Surgery, Stanford University School of Medicine, Palo Alto, California; 7 Center for Healthcare Organization and Implementation Research, VA Boston Healthcare System, Boston, Massachusetts; 8 Department of Surgery, Boston University Chobanian & Avedisian School of Medicine, Boston, Massachusetts

## Abstract

This cohort study examines the association between same-day preoperative glycemic control and postoperative adverse events among patients with diabetes undergoing ambulatory hernia surgery.

## Introduction

Hernia surgery is commonly performed with a low rate of postoperative adverse events (AEs). Risk of complications (eg, infection, bleeding, and superficial wound dehiscence) is higher for patients with type 1 or 2 diabetes,^1^ and several academic societies recommend testing glycemic control preoperatively, including the American Diabetes Association,^2^ the Society for Ambulatory Anesthesia,^3^ and the Endocrine Society.^4^ However, there is limited evidence that keeping within recommended blood glucose (BG) concentrations of 100 to 180 mg/dL (to convert to millimoles per liter, multiply by 0.0555) is associated with improved outcomes in ambulatory surgery. Our objective was to test the hypothesis that poor same-day preoperative glycemic control was associated with higher odds of postoperative AEs among Veterans Health Administration (VHA) patients with diabetes undergoing ambulatory hernia surgery.

## Methods

This study followed the STROBE reporting guideline. Our cohort included veterans who underwent hernia procedures performed between October 1, 2015, and September 30, 2019, and data were analyzed between January 10, 2022, and January 20, 2023, using SAS Enterprise Guide, version 8.3 (SAS Institute Inc). Study data were obtained from the VHA Corporate Data Warehouse. We identified all procedures by *Current Procedural Terminology* codes (eAppendix 1 in Supplement 1) and limited the cohort to patients with diabetes diagnosed with *International Statistical Classification of Diseases, Tenth Revision, Clinical Modification* codes E10 and E11 before surgery.

We created separate measures of poor preoperative diabetes control: same-day BG concentration of lower than 90 mg/dL (hypoglycemic) and same-day preoperative BG concentration of higher than 180 mg/dL (hyperglycemic). Our primary outcome was any surgical AE (wound-related complication, peritoneal abscess, bleeding, bacterial pneumonia, urinary tract infection, and sepsis) based on relevant diagnosis codes from admission, emergency department, or outpatient visit within 14 days of the procedure (eAppendix 2 in Supplement 1).^5^

We estimated a risk-adjusted logistic regression model with facility fixed effects to test the hypothesis that poor preoperative diabetes control increased the odds of 14-day AEs. Each model controlled for patient characteristics (age, sex, race, ethnicity, and comorbidities) and year of surgery. Significance was set at *P* < .05, and hypothesis tests were 2-sided. The VA Boston Healthcare System local institutional review board approved this study, which had a Health Insurance Portability and Accountability Act waiver of informed consent.

## Results

In this cohort study, we identified 13 100 hernia procedures in 137 VHA facilities among 12 654 uniquie patients (12 830 male patients [97.9%]; mean [SD] age, 66.2 [9.5] years). Descriptive statistics of the sample are presented in Table 1. Most procedures in the index sample involved men (12 830 [97.9%]) and White patients (9751 [74.4%]) (Table 1). Of 13 100 procedures, 576 (4.4%) were for patients with same-day preoperative hypoglycemia, and 1507 (11.5%) were for patients with same-day preoperative hyperglycemia; however, preoperative BG laboratory data were missing for 2464 procedures (18.8%). At least one 14-day AE occurred for 339 procedures (2.6%) (Table 1). Risk-adjusted odds of an AE were higher for patients with same-day preoperative hyperglycemia (adjusted odds ratio, 1.5 [95% CI, 1.1-2.1]) (Table 2).

**Table 1.** Sample Characteristics for Veterans Health Administration Ambulatory Hernia Procedures Among Patients With Diabetes^a^

Characteristic	Hernia procedures, No. (%)			*P* value
	Total (N = 13 100)	14-d AE (n = 339 [2.6%])	No AE (n = 12 761 [97.4%])	
Age, mean (SD), y	66.2 (9.5)	65.7 (10.0)	66.2 (9.5)	.16
Sex				
Female	270 (2.1)	11 (3.2)	259 (2.0)	.12
Male	12 830 (97.9)	328 (96.8)	12 502 (98.0)	
Race and ethnicity				
African American	2505 (19.1)	68 (20.1)	2437 (19.1)	.66
White	9751 (74.4)	245 (72.3)	9506 (74.5)	.35
Other^b^^,^^c^	267 (2)	14 (4.1)	253 (2)	.005
Race data missing	577 (4.4)	12 (3.5)	565 (4.4)	.43
Hispanic	967 (7.4)	19 (5.6)	948 (7.4)	.20
Ethnicity data missing	373 (2.9)	15 (4.4)	358 (2.8)	.08
AHRQ comorbidities				
AIDS^c^	67 (0.5)	5 (1.5)	62 (0.5)	.01
Alcohol abuse	946 (7.2)	26 (7.7)	920 (7.2)	.75
Deficiency anemias^c^	1786 (13.6)	64 (18.9)	1722 (13.5)	.004
Rheumatoid arthritis or collagen vascular diseases	229 (1.8)	6 (1.8)	223 (1.8)	.98
Chronic blood loss anemia^c^	95 (0.7)	8 (2.4)	87 (0.7)	<.001
Congestive heart failure^c^	992 (7.6)	44 (13)	948 (7.4)	<.001
Chronic pulmonary disease^c^	2411 (18.4)	97 (28.6)	2314 (18.1)	<.001
Coagulation deficiency^c^	364 (2.8)	16 (4.7)	348 (2.7)	.03
Depression^c^	2339 (17.9)	88 (26)	2251 (17.6)	<.001
Diabetes without chronic complications	11 139 (85)	290 (85.6)	10 849 (85)	.79
Diabetes with chronic complications	5918 (45.2)	166 (49)	5752 (45.1)	.16
Drug abuse^c^	654 (5)	26 (7.7)	628 (4.9)	.02
Hypertension	10 206 (77.9)	271 (79.9)	9935 (77.9)	.36
Hypothyroidism^c^	1243 (9.5)	44 (13)	1199 (9.4)	.03
Liver disease	1208 (9.2)	38 (11.2)	1170 (9.2)	.20
Lymphoma^c^	84 (0.6)	6 (1.8)	78 (0.6)	.008
Fluid and electrolyte disorders^c^	1122 (8.6)	60 (17.7)	1062 (8.3)	<.001
Metastatic cancer	122 (0.9)	6 (1.8)	116 (0.9)	.10
Other neurological disorders^c^	818 (6.2)	42 (12.4)	776 (6.1)	<.001
Obesity^c^	3349 (25.6)	108 (31.9)	3241 (25.4)	.007
Paralysis	101 (0.8)	5 (1.5)	96 (0.8)	.13
Peripheral vascular disease	1188 (9.1)	33 (9.7)	1155 (9.1)	.66
Psychoses	955 (7.3)	32 (9.4)	923 (7.2)	.12
Pulmonary circulation disease	134 (1)	7 (2.1)	127 (1)	.05
Kidney failure	1511 (11.5)	44 (13)	1467 (11.5)	.40
Solid tumor without metastasis	1057 (8.1)	29 (8.6)	1028 (8.1)	.74
Peptic ulcer disease × bleeding	88 (0.7)	2 (0.6)	86 (0.7)	.85
Valvular disease	532 (4.1)	18 (5.3)	514 (4)	.24
Weight loss	476 (3.6)	12 (3.5)	464 (3.6)	.93
Patient has continuous glucose monitor	484 (3.7)	14 (4.1)	470 (3.7)	.67
Diabetes control				
Same-day BG				
>180 mg/dL^c^	1507 (11.5)	54 (15.9)	1453 (11.4)	.009
<90 mg/dL	576 (4.4)	15 (4.4)	561 (4.4)	.98
Missing	2464 (18.8)	60 (17.7)	2404 (18.8)	.60
14-d AEs, No.^d^				
Bleeding	NA	141	NA	NA
Wound-related complication	NA	114	NA	NA
Urinary tract infection	NA	57	NA	NA
Postop sepsis	NA	26	NA	NA
Bacterial pneumonia	NA	24	NA	NA
Peritoneal abscess	NA	1	NA	NA

Abbreviations: AE, adverse event; AHRQ, Agency for Healthcare Research and Quality; BG, blood glucose; NA, not applicable.

SI conversion factor: To convert milligrams per deciliter of BG to millimoles per liter, multiply by 0.0555.

^a^ Comparisons used the χ^2^ test or the *t* test as appropriate.

^b^ Included American Indian or Alaska Native, Asian, and multiracial.

^c^ Statistically signficant at *P* < .05.

^d^ Adverse events are totals by type. Some patients had more than 1 AE; 339 patients had any AE, and a total of 363 patients had AEs that were detected.

**Table 2.** Patient Characteristics Associated With 14-Day Adverse Events Following Ambulatory Hernia Surgery^a^

Variable	Adjusted OR (95% CI)	*P* value
Patient diabetes control		
Same day BG		
≥90 and ≤180 mg/dL	1 [Reference]	NA
>180 mg/dL^b^	1.5 (1.1-2.1)	.01
<90 mg/dL	1.0 (0.6-1.7)	.90
Patient demographic charactristics		
Age, y		
26-59	1 [Reference]	NA
60-65	1.0 (0.7-1.5)	.95
66-69	1.0 (0.7-1.5)	.95
70-73	0.9 (0.6-1.3)	.53
74-97	1.1 (0.7-1.6)	.79
Sex		
Male	1 [Reference]	NA
Female	1.2 (0.6-2.4)	.65
Race and ethnicity		
All other races	1 [Reference]	NA
African American race	1.1 (0.8-1.6)	.44
Hispanic ethnicity (yes vs no)	0.9 (0.5-1.5)	.62
Patient health		
AIDS^b^	3.5 (1.3-9.2)	.001
Alcohol abuse	0.6 (0.4-1.1)	.10
Deficiency anemias	1.2 (0.8-1.7)	.41
Rheumatoid arthritis or collagen vascular diseases	0.9 (0.4-2.2)	.80
Chronic blood loss anemia^b^	2.5 (1.1-5.9)	.03
Congestive heart failure	1.3 (0.9-2.0)	.17
Chronic pulmonary disease^b^	1.6 (1.2-2.2)	<.001
Coagulation deficiency	1.1 (0.6-2.1)	.81
Depression^b^	1.5 (1.1-1.9)	.01
Drug abuse	1.3 (0.8-2.2)	.34
Hypertension	1.0 (0.7-1.4)	.97
Hypothyroidism	1.2 (0.8-1.8)	.36
Liver disease	0.9 (0.6-1.4)	.77
Lymphoma	1.9 (0.7-5.6)	.23
Fluid and electrolyte disorders^b^	1.8 (1.3-2.6)	.001
Metastatic cancer	1.5 (0.6-4.1)	.40
Other neurological disorders^b^	1.8 (1.2-2.6)	.004
Obesity	1.3 (1.0-1.7)	.07
Paralysis	1.4 (0.5-4.1)	.51
Peripheral vascular disease	0.9 (0.6-1.4)	.58
Psychoses	1.0 (0.6-1.6)	.98
Pulmonary circulation disease	1.5 (0.6-3.5)	.40
Kidney failure	0.8 (0.5-1.1)	.19
Solid tumor without metastasis	1.0 (0.6-1.6)	.94
Peptic ulcer disease × bleeding	0.8 (0.2-3.5)	.76
Valvular disease	1.0 (0.6-1.9)	.88
Weight loss	0.8 (0.4-1.6)	.56
Model characteristics		
No. of VHA facilities	134	NA
No. of procedures (No. [%] of adverse events)	10 636 (279 [2.6%])	NA
Model area under curve	0.72	NA

Abbreviations: BG, blood glucose; OR, odds ratio; NA, not applicable; VHA, Veterans Health Administration.

SI conversion factor: To convert milligrams per deciliter of BG to millimoles per liter, multiply by 0.0555.

^a^ The analysis was performed using logistic regression models with facility fixed effects. Data are from all ambulatory hernia procedures performed in the VHA between October 1, 2015, and September 30, 2019, for patients with diabetes. Insignificant temporal risk-adjustor variables are not presented.

^b^ Statistically signficant at *P* < .05.

## Discussion

We assessed the association between preoperative diabetes control and postoperative AEs in a national sample of veterans with diabetes who underwent ambulatory hernia procedures. Our complication rate was 2.6% compared with 4.7% in a Swedish registry study; this difference may be explained by our exclusion of emergent and inpatient hernia procedures.^1^ We found no association between postoperative AEs and same-day hypoglycemia; however, same-day hyperglycemia was associated with elevated risk of AE.

Our study is limited by a disproportionally White and male VA population compared with the general public, and potentially relevant clinical details were not included in risk adjustment (eg, wound class, component separation, duration of surgery, or mesh size).^6^ Future work should assess the association between diabetes control and surgical AEs outside the VHA.

The findings of this cohort study suggest that an elevated preoperative glucose level was associated with an elevated risk of AEs following ambulatory hernia surgery. Patient education on preoperative glucose management and team training to ensure glucose assessment on the day of surgery and potential intervention for hyperglycemia may present opportunities to decrease the number of postoperative AEs.

## Notes

Supplement 1. **Appendix 1.** Current Procedure Terminology (CPT) Codes for Ambulatory Hernia Surgery **Appendix 2.** International Classification of Diseases, Tenth Revision, Clinical Modification (ICD-10-CM) Codes for Surgical Complications

Supplement 2.
Data Sharing Statement
