# Supplementary material for: Association Between Preoperative Diabetes Control and Postoperative Adverse Events Among Veterans Health Administration Patients With Diabetes Who Underwent Elective Ambulatory Hernia Surgery
Source: JAMA Netw Open. 2023 Mar 31;6(3):e236318. doi: 10.1001/jamanetworkopen.2023.6318 (PMC10066455; doi:10.1001/jamanetworkopen.2023.6318)
Supplement: Supplement 1. — Appendix 1. Current Procedure Terminology (CPT) Codes for Ambulatory Hernia Surgery Appendix 2. International Classification of Diseases, Tenth Revision, Clinical Modification (ICD-10-CM) Codes for Surgical Complications [file jamanetwopen-e236318-s001.pdf]

Supplemental Online Content

Shanahan J, Vimalananda VG, Graham L, Schumann R, Mull HJ. Association between preoperative diabetes control and postoperative adverse events among Veterans Health Administration patients with diabetes who underwent elective ambulatory hernia surgery. *JAMA Netw Open*. 2023:6(3):e236318. doi:10.1001/jamanetworkopen.2023.6318

Appendix 1. Current Procedure Terminology (CPT) Codes for Ambulatory Hernia Surgery

Appendix 2. International Classification of Diseases, Tenth Revision, Clinical Modification (ICD-10-CM) Codes for Surgical Complications

This supplemental material has been provided by the authors to give readers additional information about their work.

© 2023 Shanahan J et al. *JAMA Network Open.*

Appendix 1: Current Procedure Terminology (CPT) Codes for Ambulatory Hernia Surgery

**CPT Code and Description**
49505 - Repair initial inguinal hernia; age 5 years or older; reducible
49507 - Repair initial inguinal hernia; age 5 years or older; incarcerated or strangulated
49520 - Repair recurrent inguinal hernia; any age; reducible
49521 - Repair recurrent inguinal hernia; any age; incarcerated or strangulated
49525 - Repair inguinal hernia; sliding; any age
49550 - Repair initial femoral hernia; any age; reducible
49553 - Repair initial femoral hernia; any age; incarcerated or strangulated
49555 - Repair recurrent femoral hernia; reducible
49557 - Repair recurrent femoral hernia; incarcerated or strangulated
49560 - Repair initial incisional or ventral hernia; reducible
49561 - Repair initial incisional or ventral hernia; incarcerated or strangulated
49565 - Repair recurrent incisional or ventral hernia; reducible
49570 - Repair epigastric hernia (eg; preperitoneal fat); reducible (separate procedure)
49572 - Repair epigastric hernia (eg; preperitoneal fat); incarcerated or strangulated
49585 - Repair umbilical hernia; age 5 years or older; reducible
49587 - Repair umbilical hernia; age 5 years or older; incarcerated or strangulated
49590 - Repair spigelian hernia
49600 - Repair of small omphalocele; with primary closure
49650 - Laparoscopy; surgical; repair initial inguinal hernia
49651 - Laparoscopy; surgical; repair recurrent inguinal hernia
49652 - Laparoscopy; surgical; repair; ventral; umbilical; spigelian or epigastric hernia (includes mesh insertion; when performed); reducible
49653 - Laparoscopy; surgical; repair; ventral; umbilical; spigelian or epigastric hernia (includes mesh insertion; when performed); incarcerated or strangulated
49654 - Laparoscopy; surgical; repair; incisional hernia (includes mesh insertion; when performed); reducible
49656 - Laparoscopy; surgical; repair; recurrent incisional hernia (includes mesh insertion; when performed); reducible
49659 - Unlisted laparoscopy procedure; hernioplasty; herniorrhaphy; herniotomy

© 2023 Shanahan J et al. *JAMA Network Open.*

Appendix 2. International Classification of Diseases, Tenth Revision, Clinical Modification (ICD-10-CM) Codes for Surgical Complications

Type of Complication	ICD-10-CM Diagnosis Code
**Wound Complication**	T81, K6811, K122, L03119, L03129, L03211, L03212, L03213, L03221, L03222, L03317, L03319, L03329, L03811, L03818, L03891, L03898, L0390, L0391
**Peritoneal Abscess**	K651, K353
**Hemorrhage or Hematoma**	D62, R58, T792XXA, D7801, D7802, D7821, D7822, D7831, D7832, D7833, D7834, E3601, E3602, E89810, E89811, E89820, E89821, E89822, E89823, G9731, G9732, G9751, G9752, G9761, G9762, G9763, G9764, H59111, H59112, H59113, H59119, H59121, H59122, H59123, H59129, H59311, H59312, H59313, H59319, H59321, H59322, H59323, H59329, H59331, H59332, H59333, H59339, H59341, H59342, H59343, H59349, H59351, H59352, H59353, H59359, H59361, H59362, H59363, H59369, H9521, H9522, H9541, H9542, H9551, H9552, H9553, H9554, I97410, I97411, I97418, I9742, I97610, I97611, I97618, I97620, I97621, I97622, I97630, I97631, I97638, I97640, I97641, I97648, J9561, J9562, J95830, J95831, J95860, J95861, J95862, J95863, K9161, K9162, K91840, K91841, K91870, K91871, K91872, K91873, L7601, L7602, L7621, L7622, L7631, L7632, L7633, L7634, M96810, M96811, M96830, M96831, M96840, M96841, M96842, M96843, N9961, N9962, N99820, N99821, N99840, N99841, N99842, N99843, T888XXA
**Bacterial Pneumonia**	A481, J14, J150, J151, J1520, J15211, J15212, J1529, J153, J154, J155, J156, J158, J159, J180, J182, J189
**Urinary Tract Infection**	N390, T83510A, T83511A, T83512A, T83518A
**Postoperative Sepsis**	A400, A401, A403, A408, A409, A4101, A4102, A411, A412, A413, A414, A4150, A4151, A4152, A4153, A4159, A4181, A4189, A419, A5486, B377, R571, R6520, R6521

© 2023 Shanahan J et al. *JAMA Network Open.*
