# Supplementary material for: Association Between Preoperative Diabetes Control and Postoperative Adverse Events Among Veterans Health Administration Patients With Diabetes Who Underwent Elective Ambulatory Hernia Surgery
Source: JAMA Netw Open. 2023 Mar 31;6(3):e236318. doi: 10.1001/jamanetworkopen.2023.6318 (PMC10066455; doi:10.1001/jamanetworkopen.2023.6318)
Supplement: Supplement 2. — Data Sharing Statement [file jamanetwopen-e236318-s002.pdf]

Data Sharing Statement

Shanahan. Association Between Preoperative Diabetes Control and Postoperative Adverse Events Among Veterans Health Administration Patients With Diabetes Who Underwent Elective Ambulatory Hernia Surgery. *JAMA Netw Open*. Published March 31, 2023. doi:10.1001/jamanetworkopen.2023.6318

Data
Data available: Yes
Data types: Other (please specify)
Additional Information: The datasets used and/or analyzed during the current study are available from the corresponding author on reasonable request with permission of the Veterans Health Administration.
How to access data: Contact corresponding author.
When available: With publication

Supporting Documents
Document types: Statistical/analytic code
How to access documents: Contact corresponding author.
When available: With publication

Additional Information
Who can access the data: Researchers whose proposed use of the data has been approved by the Veterans Health Administration.
Types of analyses: Determined by the Veterans Health Administration.
Mechanisms of data availability: Determined by the Veterans Health Administration.
